# Supplementary material for: Human Cytomegalovirus Oncoprotection across Diverse Populations, Tumor Histologies, and Age Groups: The Relevance for Prospective Vaccinal Therapy
Source: Int J Mol Sci. 2024 Mar 27;25(7):3741. doi: 10.3390/ijms25073741 (PMC11012084; doi:10.3390/ijms25073741)
Supplement: Supplementary file 1 [file ijms-25-03741-s001.zip › ijms-2876029-supplementary.pdf]

Supplementary Table S1. Cytomegalovirus (CMV) infection vis-à-vis the full complement of malignancies as reported by the Global Cancer Observatory (GLOBOCAN): univariate linear regression analysis with model characteristics.

Tumor/localization	*Model characteristics*		*Univariate linear regression analysis*		
	R^2	*Adjusted R^2*	*Standardized coefficients β*	*95% CI*	*p*-value
**1. Melanoma (skin)**	0.518	0.511	-0.719	-55.518 - -34.870	<0.001*
**2. Kidney**	0.627	0.622	-0.792	-24.206 - -16.731	<0.001*
**3. All cancers**	0.603	0.597	-0.776	-617.383- -418.339	<0.001*
**4. All cancers (excluding skin non-melanoma)**	0.605	0.599	-0.778	-511.328 - -347.189	<0.001*
**5. Breast**	0.569	0.563	-0.754	-157.096- -103.411	<0.001*
**6. Testis**	0.550	0.543	-0.741	-21.745 - -14.074	<0.001*
**7. Non-melanoma (skin)**	0.247	0.236	-0.497	-125.137 - -51.914	<0.001*
**8. Colorectum**	0.443	0.435	-0.665	-69.526 - -40.353	<0.001*
**9. Vulva**	0.494	0.487	-0.703	-4.388 - -2.681	<0.001*
**10. Prostate**	0.444	0.437	-0.667	-180.463- -104.942	<0.001*
**11. Corpus uteri**	0.372	0.364	-0.610	-37.289 - -19.765	<0.001*
**12. Oropharynx**	0.403	0.395	-0.635	-5.817 - -3.217	<0.001*
**13. Pancreas**	0.406	0.398	-0.638	-15.373 - -8.536	<0.001*
**14. Multiple myeloma**	0.387	0.378	-0.622	-8.010 - -4.334	<0.001*
**15. Leukemia**	0.406	0.398	-0.637	-14.086 - -7.819	<0.001*
**16. Hodgkin lymphoma**	0.389	0.380	-0.676	-19.579 - -11.543	<0.001*
**17. Non-Hodgkin lymphoma**	0.456	0.449	-0.624	-5.022 - -2.724	<0.001*
**18. Mesothelioma**	0.397	0.389	-0.630	-2.330 - -1.278	<0.001*
**19. Lip/Oral cavity**	0.134	0.122	-0.367	-8.984 - -2.243	0.001*
**20. Lung**	0.329	0.319	-0.573	-67.639 - -33.443	<0.001*
**21. Brain/CNS**	0.306	0.297	-0.554	-10.636 - -5.051	<0.001*
**22. Thyroid**	0.235	0.224	-0.485	-21.293 - -8.557	<0.001*
**23. Bladder**	0.300	0.290	-0.547	-26.023 - -12.197	<0.001*
**24. Ovary**	0.166	0.154	-0.407	-10.752 - -3.297	<0.001*
**25. Penis**	0.106	0.094	-0.326	-2.016 - -.375	0.005*
**26. Hypopharynx**	0.056	0.042	-0.236	-2.963 - -.039	0.044*
**27. Salivary glands**	0.102	0.090	-0.320	-.874 - -.154	0.006*
**28. Gallbladder**	0.070	0.057	0.265	.265 - 3.539	0.023*
**29. Nasopharynx**	0.052	0.038	0.227	-.023 - 3.451	0.053
**30. Vagina**	0.011	-0.003	-0.105	-.418 - .160	0.377
**31. Larynx**	0.014	0.000	-0.117	-3.241 - 1.089	0.325
**32. Esophagus**	0.003	-0.011	-0.052	-6.196 - 3.949	0.660
**33. Cervix uteri**	0.055	0.042	0.234	.349 - 36.440	0.046*
**34. Stomach**	0.002	-0.012	0.046	-6.684 - 9.889	0.701
**35. Kaposi's sarcoma**	0.018	0.004	0.135	-.626 - 2.321	0.255
**36. Liver**	0.034	0.021	0.185	-1.805-15.790	0.117

Supplementary Table S2. Cytomegalovirus (CMV) infection, the Human Development Index (HDI) and their relation to GLOBOCAN-reported malignancies: Multivariate linear regression analysis with model characteristics. The information originates from a set of 73 states globally.

**Tumor/localization**	**Multivariate linear regression analysis**				***Model characteristics***	
	Factor	*Stdand. coeff. β*	*95% Confidence Interval (CI)*	*p*-value	*R^2*	*Adjusted R^2*
**1. Melanoma (skin)**	CMV	-.529	-46.017 - -20.828	<0.001*	.573	.561
	HDI	.306	6.219-29.515	<0.001*		
**2. Kidney**	CMV	-.493	-16.622 - -9.031	<0.001*	.771	.765
	HDI	.484	8.128 - 15.149	<0.001*		
**3. All cancers**	CMV	-0.482	-427.382 - -220.202	<0.001*	.745	.737
	HDI	0.478	200.918-392.535	<0.001*		
**4. All cancers (excluding skin non-melanoma)**	CMV	-0.462	-338.43 - -175.618	<0.001*	.770	.763
	HDI	0.513	188.415-338.997	<0.001*		
**5. Breast**	CMV	-0.470	-110.714 - -51.989	<0.001*	.690	.681
	HDI	0.454	45.398-99.712	<0.001*		
**6. Testis**	CMV	-0.474	-15.813 - -7.165	<0.001*	.658	.648
	HDI	0.427	5.574-13.573	<0.001*		
**7. Non-melanoma (skin)**	CMV	-.372	-113.545- -19.880	0.006*	.269	.248
	HDI	.199	-10.322 - 76.308	.133		
**8. Colorectum**	CMV	-0.280	-37.341- -9.324	0.001	.696	.687
	HDI	0.632	35.699-61.61	<0.001*		
**9. Vulva**	CMV	-0.761	-4.93 - -2.74	<0.001*	0.493	0.479
	HDI	-0.103	-1.492-0.534	0.349		
**10. Prostate**	CMV	-0.470	-146.239 - -53.823	<0.001*	.494	.479
	HDI	0.308	17.813-103.286	0.006*		
**11. Corpus uteri**	CMV	-0.306	-24.353 - -4.35	0.006*	.510	.496
	HDI	0.483	11.686-30.187	<0.001*		
**12. Oropharynx**	CMV	-0.533	-5.473 - -2.137	<0.001*	0.413	0.396
	HDI	0.157	-0.505-2.581	0.184		
**13. Pancreas**	CMV	-0.250	-8.109 - -1.334	0.007*	.655	.645
	HDI	0.631	7.897-14.163	<0.001*		
**14. Multiple Myeloma**	CMV	-.338	-5.484 - -1.222	0.002*	.503	.489
	HDI	.449	2.145-6.086	<0.001*		
**15. Leukemia**	CMV	-0.218	-6.729 - -0.815	0.013*	.686	.677
	HDI	0.675	8.061-13.53	<0.001*		
**16. Hodgkin lymphoma**	CMV	-0.363	-3.597 - -0.887	0.002*	.484	.469
	HDI	0.410	1.091-3.597	<0.001*		
**17. Non-Hodgkin lymphoma**	CMV	-0.336	-11.93 5- -3.424	0.001	.627	.617
	HDI	0.539	7.457-15.329	<0.001*		
**18. Mesothelioma**	CMV	-0.344	-1.593 - -0.388	0.002*	.531	.518
	HDI	0.465	0.683 - 1.797	<0.001*		
**19. Lip/Oral cavity**	CMV	-0.368	-10.009 - -1.273	0.012*	.127	.102
	HDI	-0.019	-4.311-3.769	0.894		
**20. Lung**	CMV	-0.259	-39.905--5.328	0.011*	.580	.567
	HDI	0.574	30.388-62.367	<0.001*		

**21. Brain/CNS**	CMV	-0.219	-6.249--0.007	0.050*	.487	.472
	HDI	0.541	4.251-10.024	<0.001*		
**22. Thyroid**	CMV	-0.138	-11.466-2.901	0.238	.421	.404
	HDI	0.554	9.213-22.501	<0.001*		
**23. Bladder**	CMV	-0.328	-19.99--3.009	0.009*	.372	.354
	HDI	0.351	3.53-19.235	0.005*		
**24. Ovary**	CMV	-0.172	-7.572-1.605	0.199	.249	.228
	HDI	0.375	1.77-10.257	0.006*		
**25. Penis**	CMV	-0.251	-1.99-0.134	0.086	.110	.085
	HDI	0.112	-0.601-1.364	0.441		
**26. Hypopharynx**	CMV	-0.261	-3.566-0.232	0.084	.055	.027
	HDI	-0.048	-2.042-1.471	0.747		
**27. Salivary glands**	CMV	-0.440	-1.165 - -0.248	0.003*	.123	.098
	HDI	-0.216	-0.744-0.103	0.136		
**28. Gallbladder**	CMV	0.337	0.305-4.464	0.025*	.074	.047
	HDI	0.147	-0.958-2.888	0.320		
**29. Nasopharynx**	CMV	0.338	0.337-4.804	0.025*	.072	.045
	HDI	0.184	-0.775-3.357	0.217		
**30. Vagina**	CMV	-0.389	-0.869 - -0.23	0.007*	.153	.128
	HDI	-0.483	-0.824 - -0.133	0.001*		
**31. Larynx**	CMV	0.016	-2.623-2.929	0.913	.040	.012
	HDI	0.209	-0.769-4.366	0.167		
**32. Esophagus**	CMV	-0.181	-10.415-2.603	0.235	.028	.000
	HDI	-0.201	-10.048-1.992	0.186		
**33. Cervix uteri**	CMV	-.175	-33.474 - 5.858	0.166	.336	.317
	HDI	-.671	-67.266 - -30.889	<0.001*		
**34. Stomach**	CMV	0.172	-4.411-16.379	0.255	.037	.009
	HDI	0.244	-1.787-17.442	0.109		
**35. Kaposi's sarcoma**	CMV	0.031	-1.695-2.088	0.836	.041	.013
	HDI	-0.182	-2.812-0.686	0.230		
**36. Liver**	CMV	0.202	-3.674-18.728	0.184	.029	.001
	HDI	0.065	-8.125-12.595	0.668		
